# Curative Treatment of Severe Gram-Negative Bacterial Infections by a New Class of Antibiotics Targeting LpxC

**DOI:** 10.1128/mBio.00674-17

**Published:** 2017-07-25

**Authors:** Nadine Lemaître, Xiaofei Liang, Javaria Najeeb, Chul-Jin Lee, Marie Titecat, Emmanuelle Leteurtre, Michel Simonet, Eric J. Toone, Pei Zhou, Florent Sebbane

**Affiliations:** a Inserm, Univ. of Lille, CNRS, CHU Lille, Institut Pasteur de Lille, U1019—UMR8204—CIIL—Center for Infection and Immunity of Lille, F-59000 Lille, France; b Department of Chemistry, Duke University, Durham, North Carolina, USA; c Department of Biochemistry, Duke University Medical Center, Durham, North Carolina, USA; d Structural Biology and Biophysics Program, Duke University, Durham, North Carolina, USA; e Inserm, CHU Lille, UMR-S 1172—JPARC—Jean-Pierre Aubert Research Center, Univ. of Lille, F-59000 Lille, France; Sequella, Inc.

**Keywords:** LpxC, animal models, antimicrobial drug, antimicrobial resistance, plague

## Abstract

The infectious diseases caused by multidrug-resistant bacteria pose serious threats to humankind. It has been suggested that an antibiotic targeting LpxC of the lipid A biosynthetic pathway in Gram-negative bacteria is a promising strategy for curing Gram-negative bacterial infections. However, experimental proof of this concept is lacking. Here, we describe our discovery and characterization of a biphenylacetylene-based inhibitor of LpxC, an essential enzyme in the biosynthesis of the lipid A component of the outer membrane of Gram-negative bacteria. The compound LPC-069 has no known adverse effects in mice and is effective *in vitro* against a broad panel of Gram-negative clinical isolates, including several multiresistant and extremely drug-resistant strains involved in nosocomial infections. Furthermore, LPC-069 is curative in a murine model of one of the most severe human diseases, bubonic plague, which is caused by the Gram-negative bacterium *Yersinia pestis*. Our results demonstrate the safety and efficacy of LpxC inhibitors as a new class of antibiotic against fatal infections caused by extremely virulent pathogens. The present findings also highlight the potential of LpxC inhibitors for clinical development as therapeutics for infections caused by multidrug-resistant bacteria.

## IMPORTANCE

The rapid spread of antimicrobial resistance among Gram-negative bacilli highlights the urgent need for new antibiotics. Here, we describe a new class of antibiotics lacking cross-resistance with conventional antibiotics. The compounds inhibit LpxC, a key enzyme in the lipid A biosynthetic pathway in Gram-negative bacteria, and are active *in vitro* against a broad panel of clinical isolates of Gram-negative bacilli involved in nosocomial and community infections. The present study also constitutes the first demonstration of the curative treatment of bubonic plague by a novel, broad-spectrum antibiotic targeting LpxC. Hence, the data highlight the therapeutic potential of LpxC inhibitors against a wide variety of Gram-negative bacterial infections, including the most severe ones caused by *Y. pestis* and by multidrug-resistant and extensively drug-resistant carbapenemase-producing strains.

## INTRODUCTION

Antibiotics are key weapons in modern medicine because they save the lives of millions of patients infected with Gram-positive or -negative bacteria (1). However, the value of this armamentarium is being threatened by the alarmingly rapid development of bacterial resistance to common antimicrobial therapies, which thus poses serious threats to humankind (2). The rapid spread of antimicrobial resistance is due to horizontal gene transfer systems, such as conjugative plasmids (3). For example, horizontal gene transfer has led to the emergence of both pathogenic *Enterobacteriaceae* and opportunistic pathogens such as *Pseudomonas aeruginosa* and *Acinetobacter baumannii* that have become resistant to carbapenems—the last line of defense against multidrug-resistant (MDR) Gram-negative pathogens. Worryingly, the conjugative plasmids that confer multidrug resistance also spread among the deadliest, most pathogenic bacterial species for humans, such as the plague agent, *Yersinia pestis* (4). MDR strains of *Y. pestis* have been isolated in different parts of the world (e.g., Madagascar and Mongolia) and have thus drastically depleted the therapeutic arsenal for prophylactic and curative treatments of plague (5, 6). This is of particular concern, given that plague remains an international public health issue. Indeed, the recent upsurge of plague in the United States in 2015 and the disease’s reemergence in North Africa (Algeria and Libya) after decades of silence might herald the return of plague in Europe—especially in view of the unstable geopolitical situation worldwide (5–9). Furthermore, the emergence of MDR *Y. pestis* strains and their potential usage in bioterrorism attacks could send death tolls to levels last seen during the preantibiotic era. Hence, there is an urgent need to develop novel antibiotics against MDR Gram-negative pathogens.

Twenty years ago, the results of a study by Onishi and coworkers suggested that inhibition of LpxC, an essential cytoplasmic enzyme in the biosynthesis of lipid A in Gram-negative bacteria, was a promising strategy for countering Gram-negative bacterial infections (10). Furthermore, our previous *in vitro* investigation highlighted the therapeutic potential of LpxC inhibitors against MDR and extensively drug-resistant (XDR) Gram-negative bacilli in general and carbapenemase-producing strains in particular (11). However, neither the work of Onishi et al. nor two subsequent *in vivo* studies demonstrated that LpxC inhibitors are capable of curing an infection (12, 13). Indeed, potential relapse after the end of the treatment was not investigated, and the bacterial load in treated animals (when reported) was determined soon after the bacterial challenge (48 h, at the latest). Furthermore, previous investigations used animal models of prophylactic treatment (i.e., treatment initiated ≤60 min postchallenge) and did not assess the treatment of infections caused by highly aggressive pathogens such as *Yersinia pestis*. These limitations have profound implications for the use of LpxC inhibitors as real-world therapeutics; although extremely potent LpxC inhibitors may be able to maintain high levels of host viability during treatment, they may ultimately fail to cure the disease if they cannot completely clear the bacterial load.

Here, we report for the first time that LPC-069 (a novel biphenylacetylene-based LpxC inhibitor with activity against extended-spectrum β-lactamase [ESBL]- and carbapenemase-producing *Enterobacteriaceae*, *P. aeruginosa*, and *A. baumannii*) cures plague—one of the most severe human diseases. Our data demonstrate that (i) LpxC is a valid drug target for the treatment of common Gram-negative bacterial infections caused by MDR/XDR strains and (ii) LPC-069 is the first therapeutically attractive compound in a new class of antibiotics with activity against LpxC.

## RESULTS

### Difluoromethyl-l-*allo*-threonyl-hydroxamate LpxC inhibitors are active against *Y. pestis.*

**FIG 1:**
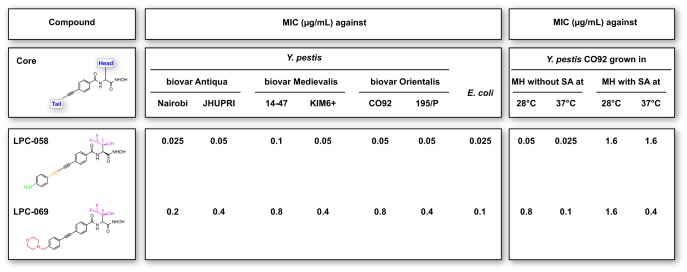
LpxC inhibitors LPC-058 and LPC-069 display antibiotic activity against *Y. pestis in vitro*. The molecular structures of LPC-058 and LPC-069, including common features and structural differences, are depicted. The MICs of LPC-058 and LPC-069 were determined for two strains of each of three *Y. pestis* biovars, grown at the optimal growth temperature (28°C) or at the host temperature (37°C) in Mueller-Hinton medium (MH) supplemented or not with serum albumin (SA). The MICs of LPC-058 and LPC-069 determined with *E. coli* grown at 37°C in MH medium are also reported. The MICs were determined from three independent biological replicates.

We recently reported on LPC-058, a difluoromethyl-l-*allo*-threonyl-hydroxamate biphenyldiacetylene-based LpxC inhibitor that displays potent antibiotic activity *in vitro* against a broad range of Gram-negative pathogens (11, 14). Likewise, LPC-058 was highly active *in vitro* against *Y. pestis* grown at either the optimal growth temperature (28°C) or the host temperature (37°C). This high level of activity was seen for the three distinct biovars of *Y. pestis* (Fig. 1). However, LPC-058’s antibacterial activity fell by a factor of 32 and 64 when *Y. pestis* was cultured at 28°C and 37°C, respectively, in the presence of 2% (wt/vol) serum albumin; this probably reflects a high level of plasma protein binding and thus low tissue penetration (Fig. 1). This observation prompted us to look for compounds that are effective against bacteria *in vitro* and are not affected (or are only slightly affected) by the presence of serum albumin. By screening our compound library with this objective in mind, we selected the morpholine-substituted biphenylacetylene molecule LPC-069 (synthesis described in Text S1 in the supplemental material), which bears the same difluoromethyl-l-*allo*-threonyl-hydroxamate head group as LPC-058 (Fig. 1). Although LPC-069 was less active than LPC-058 against *Y. pestis* grown in the absence of serum albumin at 28°C or 37°C, it was more active than LPC-058 when *Y. pestis* was grown in the presence of serum albumin at 37°C: the MIC of LPC-069 was 4-fold lower than that of LPC-058 (Fig. 1). Interestingly, we found that LPC-069 (like LPC-058) is a broad-spectrum antibiotic with activity against a wide panel of clinically relevant Gram-negative bacteria (Table S1), including MDR and XDR strains of *Enterobacteriaceae* (MIC_90_, 0.2 to 0.8 mg/liter) and *Pseudomonas aeruginosa* and *Acinetobacter baumannii* (MIC_90_, 3.2 mg/liter). Last, both LPC-058 and LPC-069 displayed a bacteriostatic effect against *Y. pestis* (Fig. S1).

10.1128/mBio.00674-17.1 TEXT S1  Large-scale synthesis of LPC-069. Download TEXT S1, PDF file, 0.1 MB. Copyright © 2017 Lemaître et al. 2017 Lemaître et al. This content is distributed under the terms of the Creative Commons Attribution 4.0 International license.

10.1128/mBio.00674-17.2 FIG S1  LPC-058 and LPC-069 show bacteriostatic activity against *Y. pestis*. Growth curves for *Y. pestis* CO92 grown at 28°C in MH medium supplemented (or not) with 0.05 to 0.4 µg/ml of LPC-058, with 8 to 6.4 µg/ml of LPC-069, or with doxycycline (Dox; used as a control at its MIC of 0.5 µg/ml) are shown. The data are representative of two independent experiments. Download FIG S1, PDF file, 0.02 MB. Copyright © 2017 Lemaître et al. 2017 Lemaître et al. This content is distributed under the terms of the Creative Commons Attribution 4.0 International license.

10.1128/mBio.00674-17.4 TABLE S1  Antimicrobial activity of LPC-069 against clinical isolates of *Enterobacteriaceae*, *Pseudomonas aeruginosa*, and *Acinetobacter baumannii*. Download TABLE S1, PDF file, 0.1 MB. Copyright © 2017 Lemaître et al. 2017 Lemaître et al. This content is distributed under the terms of the Creative Commons Attribution 4.0 International license.

### LPC-069 and LPC-058 bind to LpxC in similar manners.

**FIG 2:**
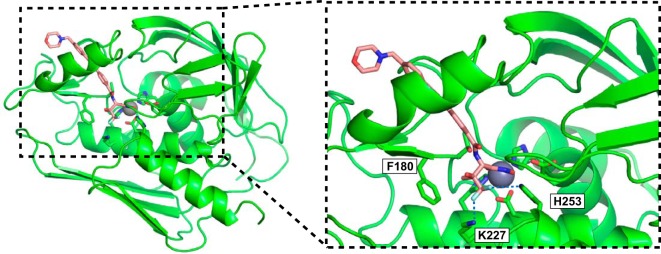
Crystal structure of LPC-069 bound to *A. aeolicus* LpxC. The structural features of LpxC are depicted as a ribbon diagram, and the catalytic zinc ion is shown as a sphere. LPC-069 and the side chains of zinc-binding residues and important catalytic residues of LpxC are shown as a stick model. Electrostatic interactions between the fluorine atoms and the positively charged K227 and H253 residues are shown as blue dashed lines (right panel).

In order to visualize the mode of binding of LPC-069 to LpxC and to compare it with that of the previously studied inhibitor LPC-058 (14), we determined LPC-069’s crystal structure in a complex with *Aquifex aeolicus* LpxC (Table S2), which is used as a model of *Y. pestis* LpxC. In the inhibitor-bound structure, LPC-069’s curved tail group occupies LpxC’s L-shaped hydrophobic substrate passage, and the terminal morpholine group adopts a chair conformation (Fig. 2). Interestingly, the crystal structure featured two different head group conformations for LPC-069. In the major conformational state (Fig. 2), the Cβ-substituted methyl group of the head group interacts with a conserved phenylalanine (F180) on LpxC, the hydroxyl group points toward the solvent, and the difluoromethyl moiety interacts with the conserved, catalytically important lysine (K227) and histidine (H253) residues in the active site. This type of interaction is consistent with that reported for LPC-058 (14). In the minor conformational state, however, the presence of a phosphate molecule in the active site causes LPC-069’s head group to undergo an ~120° rotation around its Cα-Cβ axis and thus distance itself from the protein’s residues (Fig. S2). Given the very high phosphate concentration (~1.25 M) in the crystallization buffer, the minor conformation is probably a cocrystallization artifact rather than a bona fide binding mode.

10.1128/mBio.00674-17.5 TABLE S2  Data collection and refinement statistics. Download TABLE S2, PDF file, 0.1 MB. Copyright © 2017 Lemaître et al. 2017 Lemaître et al. This content is distributed under the terms of the Creative Commons Attribution 4.0 International license.

10.1128/mBio.00674-17.3 FIG S2  Overlay of the minor- and major-state ligand conformations of LPC-069 bound to *A. aeolicus* LpxC. LpxC is shown in the ribbon diagram, and the catalytic zinc ion is shown as a sphere. LPC-069’s conformations in the minor and major states, the phosphate ion associated with the minor state, and the side chains of zinc-binding and active site residues of LpxC are shown as stick models. The locations of the head group’s Cα and Cβ atoms are indicated by brown and purple arrows, respectively. Compared with the major conformational state (in gray), the presence of a phosphate molecule in the minor conformational state (i) pushed the difluoromethyl group away from its energetically favorable position, (ii) caused an ~120° rotation around the head group’s Cα-Cβ bond, and (iii) disrupted the electrostatic interactions between the fluorine atoms and K227/H253. Download FIG S2, PDF file, 0.1 MB. Copyright © 2017 Lemaître et al. 2017 Lemaître et al. This content is distributed under the terms of the Creative Commons Attribution 4.0 International license.

### Pharmacology and toxicity of difluoromethyl-l-*allo*-threonyl-hydroxamate LpxC inhibitors.

Before testing LPC-058 and LPC-069’s efficacy against plague, we (i) assessed the stability of both compounds in the presence of hepatic microsomes and (ii) evaluated their pharmacokinetic parameters after a single intravenous administration in mice. Upon contact with hepatic microsomes, the half-life and intrinsic clearance rate were 95 min and 24 µl/min/mg for LPC-058 and 44 min and 52 µl/min/mg for LPC-069, respectively (Table S3). Hence, LPC-069 is cleared twice as quickly as LPC-058. Following the intravenous administration of a single dose of 20 mg/kg of body weight in the mouse, LPC-058 displayed a half-life of 53 min and had a moderate tissue distribution volume (1.1 liter/kg) (Table S3). After intravenous administration of a single 40-mg/kg dose, LPC-069 was found to have a shorter half-life (10 min) and a larger distribution volume (3.8 liters/kg) than LPC-058. Taken as whole, these data suggested that both molecules could be usefully tested against plague.

10.1128/mBio.00674-17.6 TABLE S3  Pharmacokinetics of the LpxC inhibitors LPC-058 and LPC-069. Download TABLE S3, PDF file, 0.1 MB. Copyright © 2017 Lemaître et al. 2017 Lemaître et al. This content is distributed under the terms of the Creative Commons Attribution 4.0 International license.

**FIG 3:**
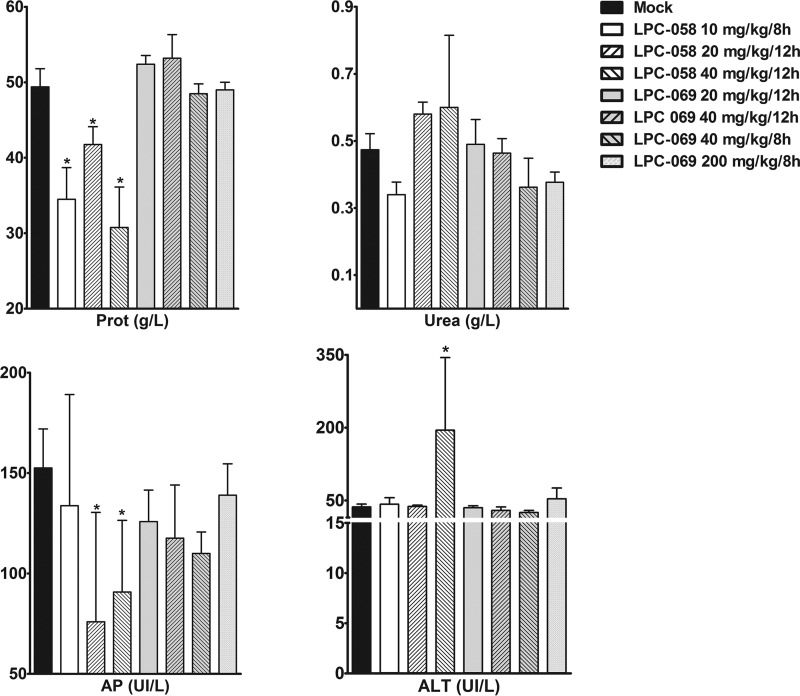
Effect of LPC-058 and LPC-069 regimens on hematologic, renal, and hepatic parameters in mice. Groups of four 8- to 9-week-old OF-1 female mice were inoculated intravenously with LPC-058 (10 mg/kg q8h, 20 mg/kg q12h, or 40 mg/kg q12h) or LPC-069 (20 mg/kg q12h, 40 mg/kg q12h, 40 mg/kg q8h, or 200 mg/kg q8h) for 5 consecutive days. On the day after the last injection, blood was collected and levels of protein (Prot), urea, alkaline phosphatase (AP), and alanine aminotransferase (ALT) were measured. The data are shown as the mean ± standard deviation. *, *P* < 0.05 in a Mann-Whitney U test.

Prior to definitively determining the compounds’ ability to protect against plague in a murine model, we assessed their toxicity and side effects. Toxicity was assessed using a 5-day regimen, which is commonly used in plague treatment models (15). Different dose levels and dosing intervals were also tested, notably because of the compounds’ relatively short half-lives. Regardless of the regimen used for LPC-058 (10 mg/kg every 8 h [q8h], 20 mg/kg q12h, or 40 mg/kg q12h), diarrhea appeared 3 days after the first injection. The diarrhea was more severe at higher doses. Low plasma protein levels were noted at the end of the 5-day regimen (Fig. 3). At the highest dose (40 mg/kg q12h), the administration of LPC-058 was associated with liver toxicity (as measured by elevated blood alanine aminotransferase level) and the accumulation of polymorphonuclear cells in the lung and large intestine (as assessed after hematoxylin-eosin staining of 5-µm sections). In contrast, mice treated with a 10 mg/kg q8h regimen displayed normal renal function (as measured by the blood urea nitrogen level) and hepatic function (Fig. 3), and no tissue damage was detected. In contrast to LPC-058, LPC-069 did not elicit any diarrhea or detectable toxicity in any of the regimens tested (including 200 mg/kg q8h) (Fig. 3).

### Efficacy of difluoromethyl-l-*allo*-threonyl-hydroxamate LpxC inhibitors in a murine model of bubonic plague.

**FIG 4:**
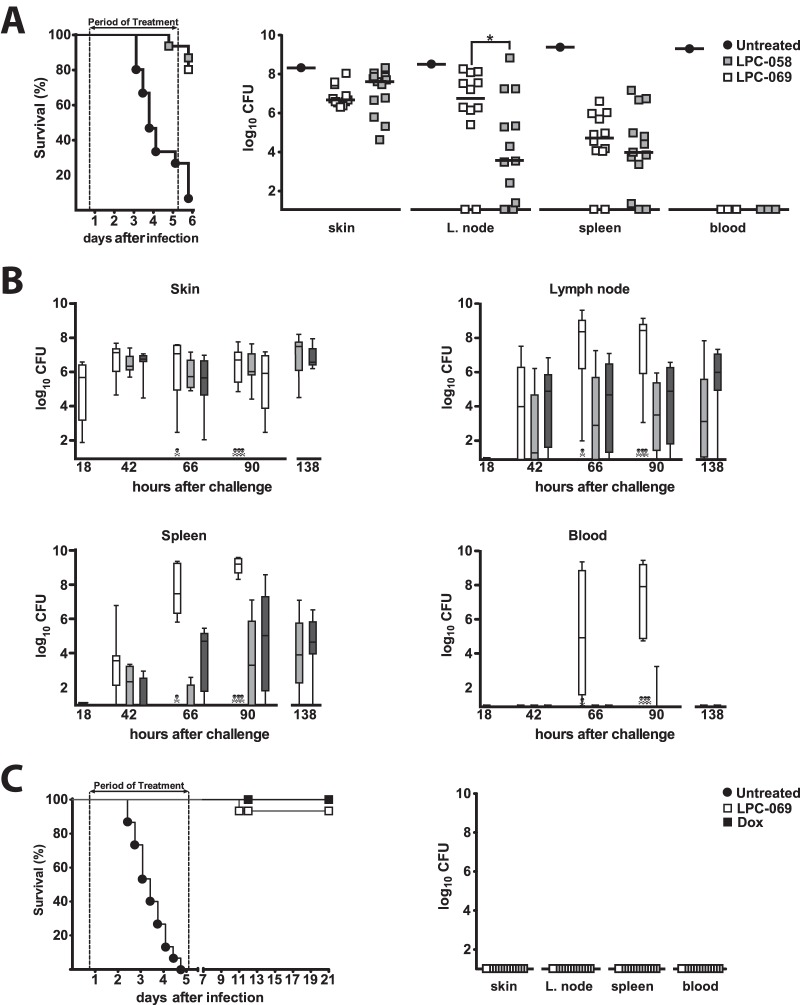
LPC-069 confers protection against plague. Eight- to 9-week-old OF-1 female mice were intradermally inoculated with 100 CFU *Y. pestis*. Eighteen hours later, mice were given a 5-day course of intravenous LPC-058 at 10 mg/kg q8h, LPC-069 at 40 mg/kg q8h (A and B) or 200 mg/kg q8h (C), or doxycycline (Dox) at 50 mg/kg q12h (C). (A) The survival of mice treated with LPC-058 (gray squares) or LPC-069 (white squares) and nontreated mice (black circles) was recorded every 8 h. The survival curves plotted for treated mice differed significantly from the curve plotted for untreated mice (*P* < 0.0001 in a log rank test). Bacterial loads in the survivors’ skin, draining lymph nodes, spleen, and blood were determined 18 h after the end of the treatment. Horizontal bars indicate the median of the individual data points. A total of 15 mice were used. (B) Bacterial loads in the skin, draining lymph nodes, spleen, and blood of eight mice at the start of treatment and in eight mice treated with LPC-058 (light gray boxes) or LPC-069 (dark gray boxes) and nontreated mice (white boxes) 24, 48, and 72 h after the start of the treatment (i.e., 42, 66, and 90 h postinfection). The bacterial loads given at the 138-h time point are those from the experiment shown in panel A. The boxes represent the interquartile range of the bacterial load, and the lines crossing the boxes indicate the median bacterial load. The whiskers represent the lowest and highest bacterial loads. Dead mice are indicated by skulls and crossbones. A two-way analysis of variance revealed that treatments with LPC-069 or LPC-058 were associated with significantly (*P* < 0.02) lower colonization of the lymph nodes, spleen, and blood, relative to nontreated controls. (C) Survival of nontreated mice (black circles) or mice treated intravenously with LPC-069 at 200 mg/kg q8h (white squares) or doxycycline (black squares) at 50 mg/kg q12h for 5 consecutive days. LPC-069 protected mice against plague as efficiently as doxycycline did (with 15 and 14 out of 15 survivors at day 21 postchallenge, respectively; *P* > 0.5). Skin, lymph nodes, spleen, and blood from all survivors were sterile. A total of 15 mice were used.

Based on the above data, 5-day regimens with LPC-058 (10 mg/kg q8h) and LPC-069 (40 mg/kg q8h) were selected for the determination of therapeutic efficacy in an established mouse model of bubonic plague, the most common form of plague, transmitted by fleas (16, 17). The mice were inoculated intradermally with 100 CFU of *Y. pestis* and then treated with an intravenous injection of LPC-058 or LPC-069 18 h after inoculation (when the bacterial load in the skin was ~10^6^ CFU). Survival was recorded every 8 h after treatment. Our data indicated that for both compounds, the survival rate was markedly higher in the treated group than in a control, nontreated group (≥80% versus ~7%, respectively [Fig. 4A]). Eighteen hours after the end of the treatment, all the survivors showed festering or signs of inflammation at the inoculation site, and most had (sometimes severe) lymphadenopathy. In agreement with the autopsy data, *Y. pestis* was isolated from the skin, draining lymph nodes, and spleen (Fig. 4A). LPC-058- and LPC-069-treated mice had similar bacterial loads in the various organs, with the exception of the lymph node, where the bacterial burden was higher in LPC-069-treated mice. The latter observation was correlated with the more severe lymphadenopathy in LPC-069-treated mice, which showed suppuration—considered to be a sign in recovery in human plague (16).

The above-described treatments with LPC-058 and LPC-069 appeared to delay colonization of the host by *Y. pestis*. This conclusion was further supported by the time courses of *Y. pestis* organ colonization in nontreated versus treated mice (Fig. 4B). The median bacterial load in the skin was ~10^6^ CFU at the start of the treatment (i.e., 18 h postchallenge) and had risen 2 days later in both treated and nontreated mice. However, the bacterial load was slightly lower in treated mice than in nontreated mice. LPC-069’s and LPC-058’s abilities to inhibit *Y. pestis* colonization were more clearly illustrated by the lower bacterial loads in the lymph nodes and (especially) in the spleen and the blood. Last, with the exception of the skin, the number of bacteria recovered after 3 days of treatment was similar to that measured after 5 days of treatment (Fig. 4B). In view of these efficacy data and the lack of any obvious adverse drug reactions for LPC-069 but not for LPC-058, we next tested the therapeutic effect of LPC-069 at a higher but nontoxic dose of 200 mg/kg q8h. Survival rates and organ sterility were assessed for up to 15 days after the end of the treatment. In contrast to nontreated mice (which all died), all the treated mice survived the 5-day treatment period, and posttreatment relapse was detected in only one of the 15 animals (Fig. 4C). An autopsy and bacteriological testing performed 2 weeks after the end of the 5-day treatment period showed that all surviving mice were free of any signs of bacterial organ colonization (Fig. 4C). Last, the LPC-069 200-mg/kg q8h regimen was found to be as efficacious as the reference treatment, the bacteriostatic compound doxycycline (Fig. 4C).

## DISCUSSION

The rapid spread of MDR Gram-negative bacteria that are resistant to the current armamentarium of antibiotics and show cross-resistance to newly developed members of preexisting antibiotic families (such as ceftolozane-tazobactam and ceftazidime- or aztreonam [Azactam]-avibactam) means that it is essential to add new, unrelated drugs to our portfolio of antimicrobial agents (18–20). LpxC inhibitors are highly attractive candidates in this context. In view of LpxC’s key role in the biosynthesis of lipid A required for integrity of the outer membrane, the testing of LpxC inhibitors has revealed potent *in vitro* antibiotic activity against a broad spectrum of Gram-negative bacilli—including those harboring mobile carbapenemase-encoding resistance genes (11). However, only three *in vivo* studies of LpxC inhibitors have been published to date, and none of them showed that LpxC inhibitors are capable of curing an infection (i.e., the absence of relapse after the end of treatment) (10, 12, 13). Furthermore, the animals in these previous studies received compounds either at the time of the infection or immediately afterward; the treatment was prophylactic, rather than curative. Last, none of the *in vivo* studies assessed the treatment of infections caused by highly aggressive pathogens, such as *Y. pestis*. Here, we showed that biphenylacetylene-based difluoromethyl-l-*allo*-threonyl-hydroxamate LpxC inhibitors, which are active against a broad range of Gram-negative bacteria, including ESBL- and carbapenemase-producing *Enterobacteriaceae*, *P. aeruginosa*, and *A. baumannii*, can cure plague in mice when first administered 18 h after the fatal infectious challenge (Fig. 4; Table S1). Hence, the treatments described here were truly curative. Our results confirmed that LpxC is a drug target for the curative treatment of common, Gram-negative bacterial infections caused by MDR/XDR strains.

In the present report, we further evaluated LPC-058—a compound already known to be highly efficient against a broad range of Gram-negative bacterial species (11)—and the novel LPC-069 molecule. LPC-058 was very active against *Y. pestis*, with a MIC similar to that of ciprofloxacin (0.03 µg/ml) (21, 22). LPC-069 was active against the same strains and species as LPC-058 but showed a lower level of activity, except when serum albumin was added to the culture medium (Fig. 1; Table S1) (11). Nonetheless, the MIC of LPC-069 against *Y. pestis* was in the same range as the values reported for gentamicin and doxycycline—both of which are recommended for treating plague (23). Similarly, LPC-069 was as active as traditional antibiotics against a large panel of clinical strains and was 4- to 8-fold more active than the new β-lactam–nonlactam combination ceftazidime-avibactam against β-lactamase-producing *P. aeruginosa* (24). In contrast to LPC-058, LPC-069 was not toxic in mice and was capable of curing plague. Although LPC-069’s half-life in mice was short (10 min), this value is similar to those reported for certain β-lactams (25). Taken as a whole, the present data suggest that LPC-069 is the first LpxC inhibitor of value for clinical development. Further optimization of LpxC inhibitors (i.e., the prolongation of *in vivo* half-lives and the reduction of serum-albumin-binding capacities) may ultimately yield a clinically safe drug for the treatment of infections caused by currently resistant “professional” or opportunistic Gram-negative pathogens.

LPC-069 and LPC-058 differed in their level of activity, both *in vitro* and *in vivo*. This difference is certainly related to the nature of the tail group, since the compounds have the same head group (Fig. 1). Indeed, the addition of a morpholine group in the acetylene- and diacetylene-based LpxC inhibitors is correlated with lower activity against Gram-negative bacilli, relative to a linear, diacetylene-based scaffold (11, 14). However, the morpholine moiety’s negative impact on activity seems to be more than counterbalanced by the weaker interaction between LPC-069’s tail group and serum albumin; LPC-069 has a higher overall level of activity than LPC-058 in the presence of serum albumin *in vitro* and presumably has a greater volume distribution *in vivo*. The exact reason for the weaker interaction between LPC-069 and serum albumin remains to be characterized. We hypothesize that LPC-069’s lack of toxicity is related to the replacement of the amine group on LPC-058’s phenyl group (i.e., the aniline group) with a morpholine group. Indeed, LPC-058 toxicity was probably due to this aniline group, which is reportedly associated with liver toxicity (26, 27). Last, it is also possible that the differential toxicity of LPC-058 versus LPC-069 in mice may be related to distinct drug exposure, as the area under the concentration-time curve (AUC) of LPC-058 following a single dose of 20 mg/kg is approximately 10-fold greater than the AUC for LPC-069 with a single dose of 40 mg/kg. Overall, the data indicate that subsequent efforts to optimize LpxC inhibitors such as LPC-069 should be focused on the tail group.

Last, we found that LPC-069 and LPC-058’s MICs against *Y. pestis* were higher at 28°C than at 37°C. This may be due to structural differences in *Y. pestis*’ lipooligosaccharide (LOS) at these temperatures; at 28°C, the phosphate groups in *Y. pestis*’ lipid A are glycosylated with 4-aminoarabinose—thus resulting in a more closely packed LOS layer and a less fluid outer membrane (28, 29). This lack of fluidity at 28°C might block the access of hydrophobic LpxC inhibitors to the cytoplasmic LpxC enzyme. In other words, our data indicate that a MIC should be measured at the host temperature (rather than the optimal growth temperature) when the structure of the bacterium’s LOS or lipopolysaccharide is known to be affected by the growth temperature.

In conclusion, we showed that LpxC inhibitors constitute a new class of antibiotics that may be capable of treating a number of infectious diseases, including the most severe ones such as plague, and infections caused by MDR/XDR carbapenemase-producing strains. The LpxC inhibitor LPC-069 is likely to be of value for clinical development and could probably be optimized for that purpose, notably via the modification of its tail group.

## MATERIALS AND METHODS

### Bacterial strains.

Clinical isolates of *Y. pestis* biovars Antiqua (JUPHRI and Nairobi), Medievalis (KIM and 1447), and Orientalis (CO92 and 195/P); *Escherichia coli* (*n* = 20); *Klebsiella pneumoniae* (*n* = 22); *Enterobacter* spp. (*n* = 20); *Proteus mirabilis* (*n* = 20); *Citrobacter koseri* (*n* = 25); *Citrobacter freundii* (*n* = 12); *Serratia marcescens* (*n* = 20); *Morganella morganii* (*n* = 23); *Yersinia* spp. (*n* = 20, including 10 *Y. enterocolitica* and 10 *Y. pseudotuberculosis* isolates); *Shigella* spp. (*n* = 21); *Salmonella enterica* (*n* = 22); *Pseudomonas aeruginosa* (*n* = 51); and *Acinetobacter baumannii* (*n* = 25) were used in this study. Seventy-seven of these strains are MDR or XDR, according to the interim standard definitions for acquired resistance (30). With the exception of *Y. pestis* strains, all bacterial strains were isolated from patient samples at Lille University Hospital (Lille, France). Last, *E. coli* ATCC 25922, *P. aeruginosa* ATCC 27853, and *A. baumannii* ATCC 17978 were used as quality controls for antimicrobial assays.

### LpxC inhibitors.

LPC-058 was synthesized as previously described (31). The synthesis of LPC-069 is described in Text S1 in the supplemental material. LpxC inhibitors were dissolved in dimethyl sulfoxide (DMSO) (for *in vitro* studies) or in saline containing hydroxypropyl-beta-cyclodextrin (Kleptose HPB; Roquette, Lille, France) (for *in vivo* studies).

### Crystal structure analysis of LPC-069 bound to *A. aeolicus* LpxC.

A protein sample of *A. aeolicus* LpxC containing residues 1 to 275 and a C181A mutation was prepared as described previously (32). The purified sample was incubated with a 4-fold molar excess of LPC-069 for 20 min at room temperature; this protein-inhibitor mixture contained 0.5 mg/ml LPC-069 and 8 mg/ml protein in 100 mM potassium chloride, 25 mM HEPES (pH 7.0), 2 mM dithiothreitol, 50 μM zinc sulfate, and 1% dimethyl sulfoxide (DMSO). The protein-inhibitor mixture was then mixed with an equivalent volume of crystallization reservoir solution containing 2.49 M diammonium hydrogen phosphate, 0.1 M Tris (pH 7.7), and 5% glycerol. LpxC-LPC-069 complex crystals were obtained with the sitting-drop vapor diffusion method at 20°C. Crystals were harvested and cryoprotected using the mother liquor solution containing 30% ethylene glycol, prior to flash-freezing. Diffraction data were collected at the SER-CAT 22-ID beamline at the Advanced Photon Source at Argonne National Laboratory and were processed with XDS software (33). The crystal structure of the *A. aeolicus* LpxC/LPC-069 complex was solved by molecular replacement using the Phaser program (34) and with Protein Data Bank (PDB) entry 5DRO as the search model. The inhibitor’s restraints were generated with eLBOW (35) and then edited manually. Iterative model building and refinement were carried out using Coot (36) and PHENIX (37). The coordinates of the *A. aeolicus* LpxC/LPC-069 complex have been deposited in the PDB (accession code 5U86).

### *In vitro* susceptibility testing of LpxC inhibitors.

MIC values were determined by using the broth microdilution method in 96-well plates containing Mueller-Hinton (MH) broth (supplemented or not with 2% bovine serum albumin [Sigma-Aldrich]) and the agar dilution method, according to the Clinical and Laboratory Standards Institute’s guidelines (38). The final concentration of LpxC inhibitor ranged from 0.012 to 6.4 μg/ml. Plates were incubated for 48 h at 28°C and 37°C for *Yersinia* species and for 24 h at 37°C for the other organisms. The MIC was defined as the lowest concentration at which no visible growth occurred. All assays were performed in triplicate on different days (three biological replicates). For the kill-curve assay, 5 × 10^5^ CFU/ml of *Y. pestis* CO92 was incubated at 28°C with shaking in MH broth supplemented (or not) with different concentrations (1×, 4×, or 8× MIC) of LPC-069 or LPC-058 or with 0.5 µg/ml of doxycycline (corresponding to doxycycline’s MIC for *Y. pestis* CO92 strain) (15). Samples were collected after 0, 3, 6, 10, and 24 h of incubation; serially diluted; and then spread onto blood agar plates to determine bacterial counts (CFU). Bactericidal activity was defined as a ≥3-log_10_ killing effect. Time-kill curves were determined twice on different days (two biological replicates). The assay’s limit of detection (LOD) was ≤1.3 log_10_ CFU/ml.

### Microsomal stability analysis.

The metabolic stability analysis of LpxC inhibitors was performed with mouse liver microsomes (BD Gentest, Le Pont de Claix, France) at 37°C. The reaction mixture contained microsomal protein (0.3 mg/ml), 1 µM LPC compound in 1% methanol vehicle, 5 mM MgCl_2_, 1 mM NADP, 5 mM glucose-6-phosphate, 0.4 U/ml glucose-6-phosphate dehydrogenase, and 50 mM potassium phosphate buffer (pH 7.4). Samples were taken at 5, 10, 20, 30, and 40 min, and the reaction was terminated by adding 4 volumes of an ice-cold acetonitrile internal standard. After the removal of precipitated protein by centrifugation, the amount of intact compound in the supernatant was quantified by high-performance liquid chromatography–tandem mass spectrometry (LC-MS/MS) (39).

### *In vivo* pharmacokinetics.

LPC-058 and LPC-069 were administered intravenously to groups of 27 female OF-1 mice (aged 8 to 9 weeks) at a dose level of 20 and 40 mg/kg, respectively. At 0, 5, 10, 20, 30, 60, 120, 240, and 480 min after the injection, three mice were sacrificed, and the blood was immediately collected by cardiac puncture. The plasma level of the tested LpxC inhibitor was determined by LC-MS/MS (39).

### Tolerability studies in mice.

Tolerability in groups of up to 10 female OF-1 mice (aged 8 to 9 weeks) was determined during a 5-day course of treatment. During this period, vomiting, diarrhea, seizures, irritability, and dazed state were scored as signs of general toxicity. At the end of the course of treatment, cardiac blood was collected in order to detect putative renal and hepatic dysfunction. Proteinemia and renal and hepatic function were determined by measuring blood urea nitrogen, alanine aminotransferase, and alkaline phosphatase levels, respectively. Last, a histological analysis of the livers, kidneys, small and large intestines, lungs, and brains from three treated mice and three nontreated mice was used to identify possible tissue damage. Tissues were fixed in 10% neutral buffered formalin, processed in paraffin blocks (using standard techniques), cut into 5-µm sections on a microtome, and then stained with hematoxylin-eosin reagent on glass slides.

### Treatment of plague in a murine model.

Female OF-1 mice (aged 8 to 9 weeks) were intradermally inoculated in the upper right thigh with 100 CFU of *Y. pestis* grown overnight at 21°C. Eighteen hours after inoculation, mice were randomly assigned (by the manipulator assisting the person who injected animals) and then injected intravenously (*n* = 15) with 20% HPB-cyclodextrin in saline (in the control group), doxycycline 50 mg/kg q12h (in the reference treatment group), or different doses of LpxC inhibitors (in the investigational group). Survival was noted every 8 h until the end of the 5-day course of treatment and then 18 h and 15 days after the injection of the last dose.

### Assessment of efficacy.

Treated and nontreated female OF-1 mice (aged 8 to 9 weeks) were sacrificed 18, 42, 66, and 90 h after challenge (i.e., during treatment) and then 18 h and 15 days after the last injection (i.e., after the end of the treatment). Immediately after sacrifice, cardiac blood, skin from around the injection site, inguinal and axillary lymph nodes, and the spleen were aseptically collected. The skin biopsy specimen was lysed by incubation in 1.5 ml sterile phosphate-buffered saline (PBS) containing 2.4 U of collagenase/dispase for 60 min at 37°C. It was then triturated using the FastPrep FP120 instrument (Qbiogene). The lymph nodes and the spleen were triturated through sterile mesh into 3 ml of sterile, cold PBS and then homogenized. Triturated organs and heart blood were serially diluted into PBS, and the CFU was counted on agar blood plates. Tissues containing <1.8 log_10_ CFU (i.e., below the LOD) were considered to be sterile.

### Statistical analysis.

A two-way analysis of variance, the log rank test, and the Mann-Whitney U test were used to analyze the results of growth in infected tissues, survival curves, and tolerability studies in mice. The MICs and the kill curve were determined from three and two independent biological replicates, respectively.

### Ethics statement.

Animals were housed at the Institut Pasteur de Lille animal facility (Lille, France), which is accredited by the French health authorities for the performance of experiments on live rodents. It complies with French and European regulations on the care and protection of laboratory animals. The animal experiments complied with current national and institutional regulations and ethical guidelines and were approved by the local Institutional Animal Care and Use Committee.

### Data availability.

The coordinates of the *A. aeolicus* LpxC/LPC-069 complex have been deposited in the PDB (accession code 5U86). Other data that support the findings of this study are available from the corresponding authors upon reasonable request.
