# Supplementary material for: Curative Treatment of Severe Gram-Negative Bacterial Infections by a New Class of Antibiotics Targeting LpxC
Source: mBio. 2017 Jul 25;8(4):e00674-17. doi: 10.1128/mBio.00674-17 (PMC5527309; doi:10.1128/mBio.00674-17)
Supplement: TEXT S1 [file mbo004173392s1.pdf]

Supplementary Data 1. Large-scale synthesis of LPC-069.

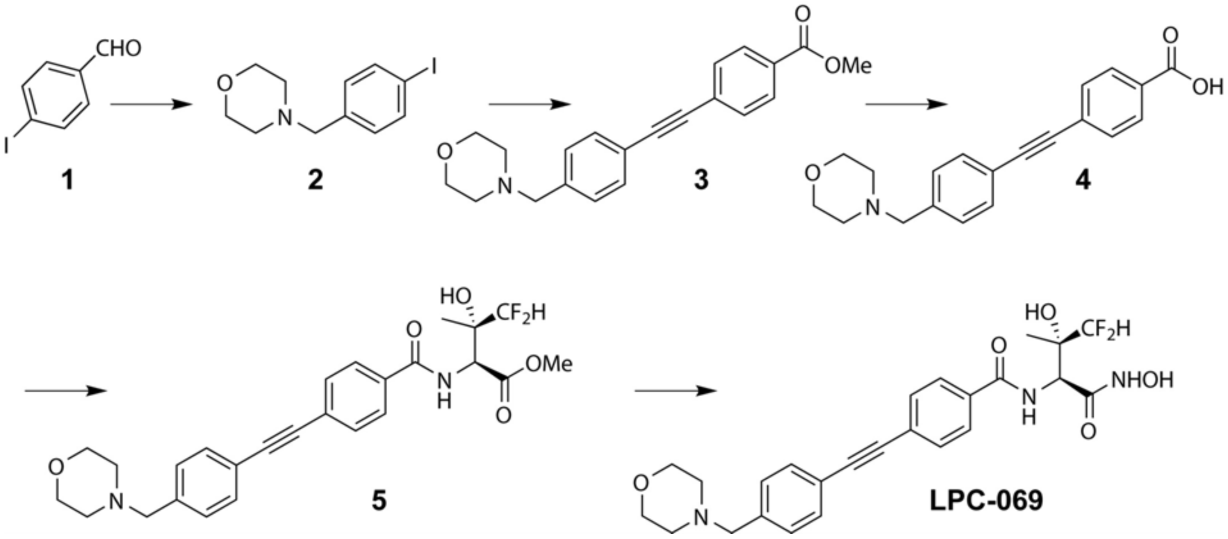

4-(4-Iodobenzyl)morpholine(2): To a solution of 4-iodobenzaldehyde (**1**) (2.67 g, 11.5 mmol, 1.00 equiv) in anhydrous THF (50 mL) was added morpholine (1.25 g, 14.4 mmol, 1.25 equiv) at room temperature under argon. The mixture was then cooled to 0 °C. 4.87 g of $NaHB(OAc)_3$ was added in four portions. The reaction mixture was stirred at 0 °C for 1 hr, and then was allowed to warm to room temperature for 15 hrs. The yellow suspension mixture was concentrated to dryness. The residue was diluted with water (100 mL), extracted with EtOAc (3×50 mL). The combined organic layers were washed with water (50 mL) and brine (50 mL), and then dried with Na_2SO_4. Evaporation of the solvent afforded the crude product, which was purified by flash chromatography (eluting with EtOAc in hexane 0-50%) to give the product as white solid (3.17 g, yield 91%). ^{1}H NMR (300 MHz, $CDCl_3$): δ 2.41 (t, 4H, J=9.3 Hz), 3.42 (s, 2H), 3.69 (t, 4H, J=9.3 Hz), 7.08 (d, 2H, J=8.4 Hz), 7.63 (d, 2H, J=8.4 Hz); ^{13}C (75 MHz, $CDCl_3$): δ 53.80, 63.00, 67.21, 92.71, 131.31, 137.59, 137.87; HPLC-MS (ESI, m/z): 303.1429 $[M+H]^+$.

Methyl 4-((4-(morpholinomethyl)phenyl)ethynyl)benzoate (3): To a solution of compound **2** (1.81 g, 6.0 mmol, 1.20 equiv) in anhydrous THF (20 mL) was added methyl 4-ethynylbenzoate (0.8 g, 5.0 mmol, 1.00 equiv), $Pd(Cl_2(PPh_3)_2$ (0.35 g, 0.5 mmol, 0.10 equiv), PPh_3 (0.066 g, 0.25 mmol, 0.005 equiv), CuI (0.048 g, 0.25 mmol, 0.05 equiv) and TEA (2.1 mL, 15.0 mmol, 3.0 equiv). The reaction mixture was then heated to 45 °C under argon for 14 hrs. The suspension mixture was concentrated to dryness. The residue was diluted with water (100 mL), extracted with EtOAc (3×50 mL). The combined organic layers were washed with water (50 mL) and brine (50 mL), and dried with Na_2SO_4. Evaporation of the solvent afforded the crude product, which was purified by flash chromatography (eluting with EtOAc in hexane 0-50%) to give the product as white solid (1.17 g, yield 70%). ^1H NMR (400 MHz, $CDCl_3$): δ 2.41 (br, s, 4H), 3.47 (s, 2H), 3.68 (t, 4H, J=8.8 Hz), 3.89 (s, 3H), 7.30 (d, 2H, J=8 Hz), 7.47 (d, 2H, J=8 Hz), 7.55 (d, 2H, J=10.4 Hz), 7.99 (d, 2H, J=10.4 Hz); ^{13}C (100 MHz, $CDCl_3$): δ 52.19, 53.60, 63.09, 66.96, 88.52, 92.31, 121.42, 128.01, 129.13, 129.39, 129.49, 131.44, 131.64, 138.85, 166.49; HPLC-MS (ESI, m/z): 335.4036 $[M+H]^+$.

4-((4-(Morpholinomethyl)phenyl)ethynyl)benzoic acid (4): To a solution of methyl ester **3** (0.73 g, 2.2 mmol, 1.0 equiv) in methanol (8 mL) and H_2O (4 mL) was added $LiOH.H_2O$ (0.097 g, 2.3 mmol, 1.05 equiv). The reaction mixture was heated to reflux for 3 hrs under argon. The resulting mixture was concentrated to dryness. The residue was diluted with 30 mL of water and added 1N HCl to pH around 3. The white precipitate was filtered and dried to afford the compound as white solid (0.66 g, yield 93%). ^1H NMR

(300 MHz, DMSO-d_6): δ 2.63 (br, s, 4H), 3.66 (br, s, 4H), 3.80 (s, 2H), 7.47 (d, 2H, J=7.6 Hz), 7.55 (d, 2H, J=7.6 Hz), 7.62 (d, 2H, J=8.4 Hz), 7.93 (d, 2H, J=8.4 Hz); ^{13}C (75 MHz, DMSO-d_6): δ 53.60, 63.09, 66.96, 88.52, 92.31, 121.42, 128.01, 129.13, 129.39, 129.49, 131.44, 131.64, 138.85, 166.49; HPLC-MS (ESI, m/z): 322.1469[M+H]$^+$.

(2*S*, 3*S*)-methyl-4,4-difluoro-3-hydroxy-3-methyl-2-(4-((4 (morpholinomethyl) phenyl)ethynyl)benzamido)butanoate (5): To a solution of acid **4** (73 mg, 0.228 mmol,1.00 equiv) in anhydrous DMF (1 mL) was added amino acid methyl ester (31) (93 mg, 0.273 mmol, 1.20 equiv), EDC (52 mg, 0.273 mmol, 1.20 equiv), HOBt (37 mg, 1.20 equiv, 37 mmol) and DIPEA(0.24 mL, 1.14 mmol, 5.0 equiv) at room temperature under argon. The reaction mixture was stirred at room temperature for 14 hrs. The resulting mixture was concentrated to dryness. The residue was diluted with water (20 mL), extracted with EtOAc (3×50 mL). The combined organic layers were washed with water (50 mL) and brine (50 mL), and then dried with Na_2SO_4. Evaporation of the solvent afforded the crude product, which was purified by flash chromatography (eluting with MeOH in DCM 0-3%) to provide the product as white solid (95 mg, yield 86%). ^1H (300MHz, $CDCl_3$): δ 1.38 (s, 3H), 2.44 (t, 4H, J=9 Hz), 3.50 (s, 2H), 3.70 (t, 4H, J=9 Hz), 3.81 (s, 3H), 5.03 (d, 1H, J=9 Hz), 5.77 (t, 1H, J=55.6 Hz), 7.04 (d, 1H, J=9 Hz), 7.32 (d, 2H, J=8.1 Hz), 7.49 (d, 2H, J=7.8 Hz), 7.58 (d, 2H, J=7.8 Hz), 7.79 (d, 2H, J=8.1 Hz); ^{13}C (75 MHz, $CDCl_3$): δ 18.08, 52.92, 53.58, 55.98, 63.08, 66.96, 74.04, 88.31, 92.2, 105.00, 115.78, 127.27, 127.48, 127.82, 129.16, 131.66, 131.77, 132.25, 138.83, 167.06, 170.22; ^{19}F (300 MHz, $CDCl_3$): δ -129.26, -134.68 (ABq, J_{HH}=49.8 Hz, J_{HF}=180.9 Hz); HPLC-MS (ESI, m/z): 487.2373 [M+H]$^+$.

***N*-((2*S*,3*S*)-4,4-difluoro-3-hydroxy-1-(hydroxyamino)-3-methyl-1-oxobutan-2-yl)-4-((4-(morpholinomethyl)phenyl)ethynyl)benzamide (LPC-069):** To a solution of methyl ester **5** (84 mg, 0.18 mmol, 1.0 equiv) in methanol (4 mL) was added KCN (10 mg) at 0 °C under argon. Then 2 mL of 50% NH_2OH was added. The reaction mixture was stirred at 0 °C for 2 hrs, and then was allowed to warm to room temperature for 14 hrs. The resulting mixture was concentrated to dryness. The residue was diluted with water (100 mL), extracted with EtOAc(3×30 mL). The combined organic layers were washed with water (50 mL), brine (50 mL) and dried (Na_2SO_4). Evaporation of the solvent afforded the crude product, which was purified by flash chromatography (eluting with MeOH in DCM 0-10%) to give the product as white solid (63 mg, yield 72%). ^1H (300 MHz, CD_3OD): δ 1.36 (s, 3H), 2.47 (t, 4H, J=9Hz), 3.55 (s, 2H), 3.69 (t, 4H, J=9 Hz), 4.73 (s, 1H), 5.80 (t, 1H, J=56.2 Hz), 7.38 (d, 2H, J=8.1 Hz), 7.51 (d, 2H, J=8.1 Hz), 7.62 (d, 2H, J=8.1 Hz), 7.86 (d, 2H, J=8.1 Hz); ^{13}C (75MHz, CD_3OD): δ 17.77, 53.61, 55.25, 62.43, 66.63, 72.94, 88.94, 91.94, 116.35, 120.87, 125.91, 128.42, 129.66, 131.67, 131.84, 133.86, 139.73, 165.24, 165.96; ^{19}F (300MHz, CD_3OD): δ -127.98, -136.07(ABq, J_{HH}=30.3 Hz, J_{HF}=177.8 Hz); HRMS: $C_{27}H_{27}F_2N_3O_5$ calculated 487.1919, obtained 487.1925.
