# Supplementary figures and images for: Curative Treatment of Severe Gram-Negative Bacterial Infections by a New Class of Antibiotics Targeting LpxC
Source: mBio. 2017 Jul 25;8(4):e00674-17. doi: 10.1128/mBio.00674-17 (PMC5527309; doi:10.1128/mBio.00674-17)

Supplementary Figure 1

**A**

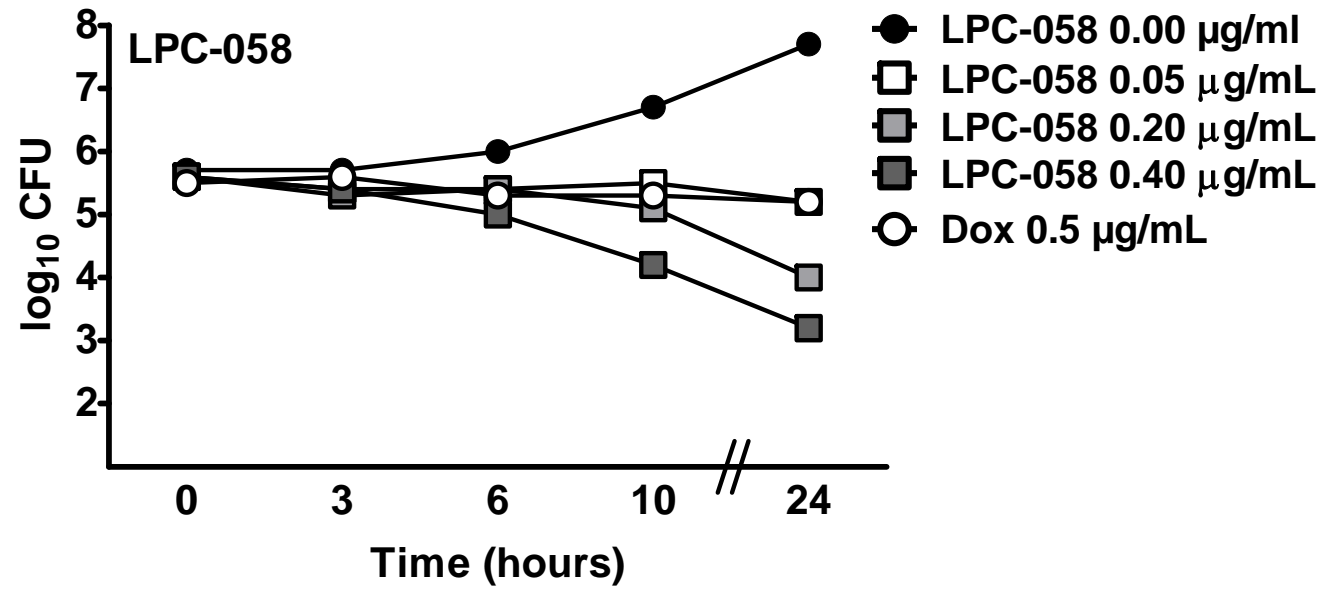

**B**

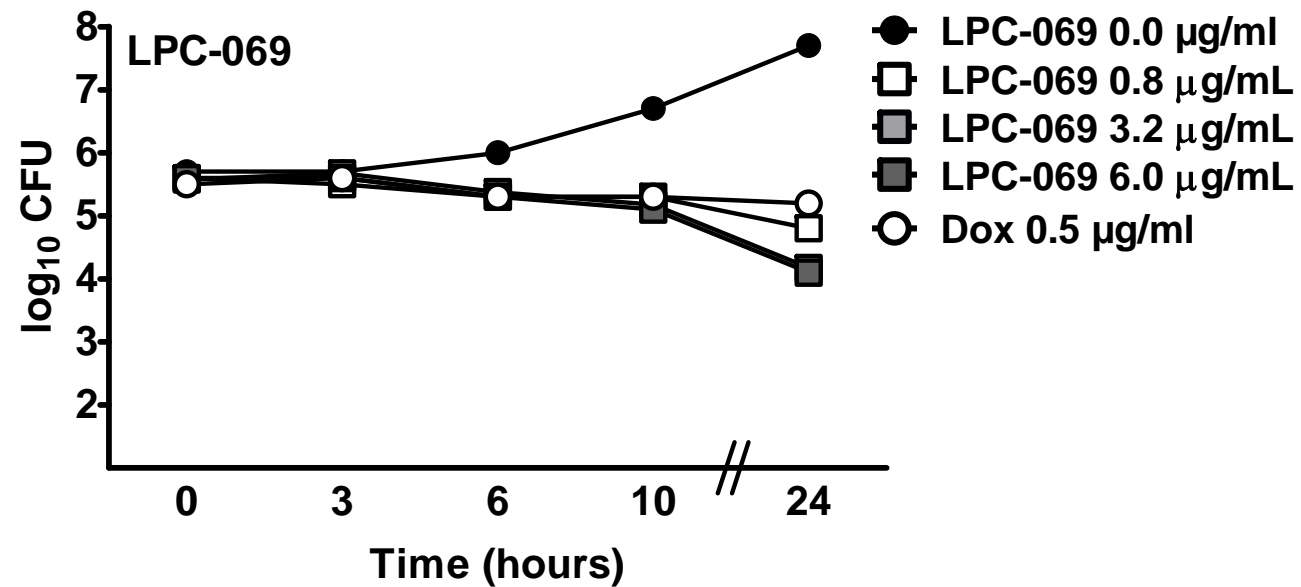

Supplement: FIG S1 [file mbo004173392sf1.pdf]

Supplementary Figure 2

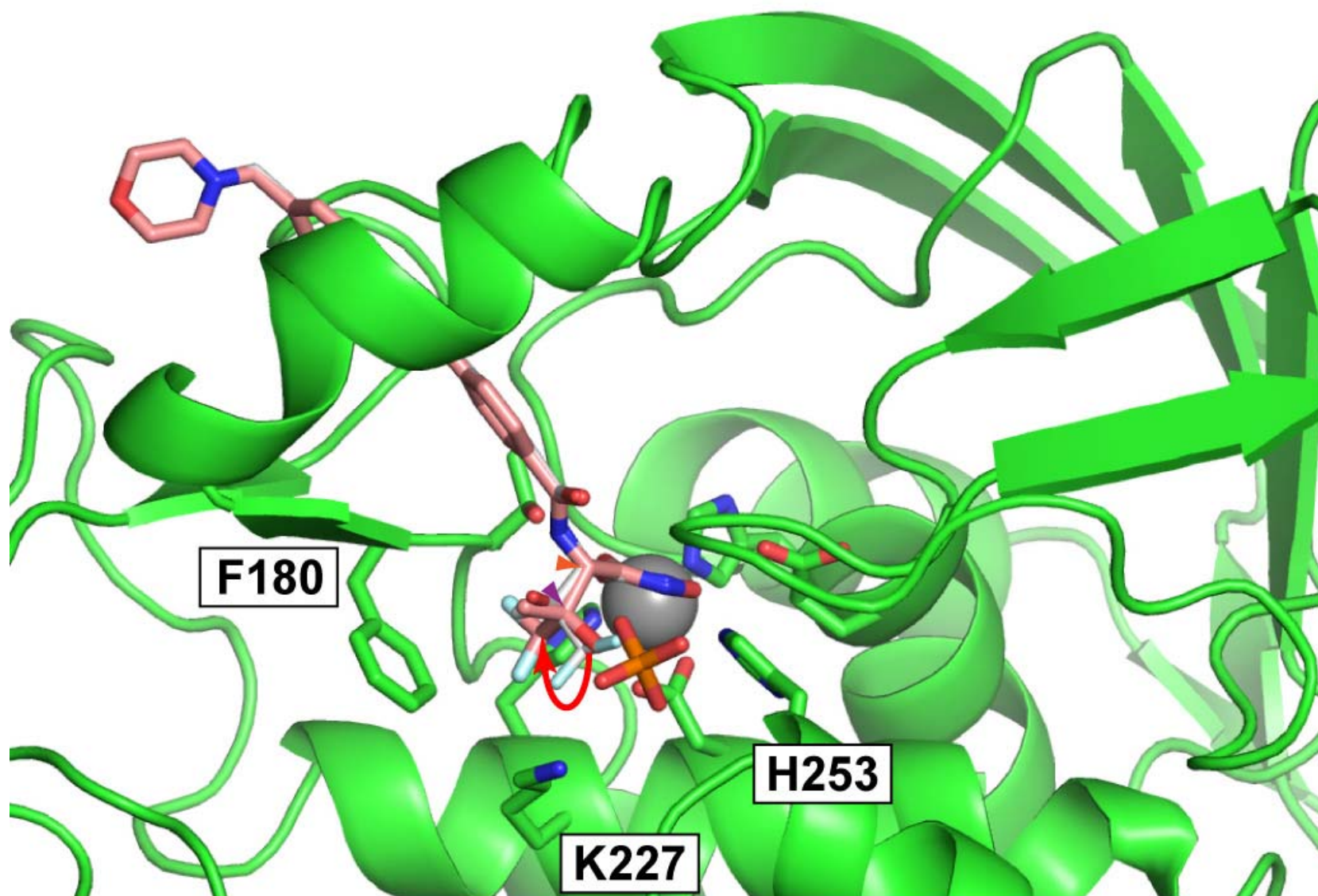

Supplement: FIG S2 [file mbo004173392sf2.pdf]
