# Supplementary material for: Curative Treatment of Severe Gram-Negative Bacterial Infections by a New Class of Antibiotics Targeting LpxC
Source: mBio. 2017 Jul 25;8(4):e00674-17. doi: 10.1128/mBio.00674-17 (PMC5527309; doi:10.1128/mBio.00674-17)
Supplement: TABLE S1 [file mbo004173392st1.pdf]

**Supplementary Table 1.** Antimicrobial activity of LPC-069 against clinical isolates of *Enterobacteriaceae*, *Pseudomonas aeruginosa* and *Acinetobacter baumannii*.

| Organism group (number of isolates tested) | MIC (mg/L) | LPC-069 |
|---|---|---|
| *E. coli* (n=20) | MIC 50 | 0.1 |
| | MIC 90 | 0.2 |
| | Range | 0.05-0.4 |
| *K. pneumoniae* (n=22) | MIC 50 | 0.4 |
| Including 15 MDR/XDR strains [a] | MIC 90 | 0.8 |
| | Range | 0.2-0.8 |
| *Enterobacter.* spp (n=20) | MIC 50 | 0.2 |
| Including 9 MDR/XDR strains | MIC 90 | 0.4 |
| | Range | 0.1-1.6 |
| *P. mirabilis* (n=20) | MIC 50 | 0.2 |
| | MIC 90 | 0.4 |
| | Range | 0.2-0.8 |
| *C. koseri* (n=25) | MIC 50 | 0.4 |
| | MIC 90 | 0.4 |
| | Range | 0.1-1.6 |
| *C. freundii* (n=12) | MIC 50 | 0.2 |
| Including 2 MDR/XDR strains | MIC 90 | 0.4 |
| | Range | 0.2-0.4 |
| *S. marcescens* (n=20) | MIC 50 | 0.2 |
| Including 5 MDR/XDR strains | MIC 90 | 0.2 |
| | Range | 0.1-0.4 |
| *M. morganii* (n=23) | MIC 50 | 0.2 |
| Including 7 MDR/XDR strains | MIC 90 | 0.8 |
| | Range | 0.2-1.6 |
| *Yersinia* spp. (n=20) | MIC 50 | 0.4 |
| | MIC 90 | 0.8 |
| | Range | 0.2-0.8 |
| *Shigella* spp. (n=21) | MIC 50 | 0.05 |
| | MIC 90 | 0.1 |
| | Range | 0.025-0.2 |
| *Salmonella* spp (n=22) | MIC 50 | 0.4 |
| | MIC 90 | 0.4 |
| | Range | 0.2-0.8 |
| *P. aeruginosa* (n=51) | MIC 50 | 1.6 |
| Including 20 MDR/XDR strains [b] | MIC 90 | 3.2 |
| | Range | 0.2-3.2 |
| *A. baumannii* (n=25) | MIC 50 | 0.8 |
| Including 19 MDR/XDR strains [c] | MIC 90 | 3.2 |
| | Range | 0.5-≥6.4 |

[a], includes five carbapenemase-producing strains (1 KPC-2, 1 NDM-1 and 3 OXA-48); [b], includes five carbapenemase-producing strains (4 VIM and one IMP); [c], includes eight carbapenemase-producing strains (7 OXA-23 strains and one OXA-40 strains)
