# Supplementary material for: Curative Treatment of Severe Gram-Negative Bacterial Infections by a New Class of Antibiotics Targeting LpxC
Source: mBio. 2017 Jul 25;8(4):e00674-17. doi: 10.1128/mBio.00674-17 (PMC5527309; doi:10.1128/mBio.00674-17)
Supplement: TABLE S2 [file mbo004173392st2.pdf]

Supplementary Table 2. Data collection and refinement statistics

	A. aeolicus **LpxC/LPC-069**
**Data collection**	
Wavelength	1.0000 Å
Space group	P 61
Cell dimensions	
a, *b*, *c* (Å)	65.99, 65.99, 132.42
α, β, γ (°)	90.0, 90.0, 120.0
Resolution (Å)	50.0 – 1.62 (1.66 – 1.62)
R_{meas}	9.7 (76.2)
$I / \sigma I$	20.13 (4.95)
Completeness (%)	100 (100)
Redundancy	12.4 (12.4)
**Refinement**	
Resolution (Å)	29.53 – 1.62 (1.66 – 1.62)
Unique reflections	41379 (2956)
Redundancy	12.4 (12.4)
R_{work} / R_{free}	0.139/0.154
No. atoms	2869
protein	2323
ligand/ion	98
water	448
Average *B*-factors	18.29
protein	15.36
ligand/ion	19.49
water	33.19
R.m.s. deviations	
bond lengths (Å)	0.006
bond angles (°)	0.90
Ramachandran	
Favored (%)	96.9
allowed (%)	3.1
outliers (%)	0.0

*Values in parentheses correspond to the highest-resolution shell.
