# Supplementary material for: Curative Treatment of Severe Gram-Negative Bacterial Infections by a New Class of Antibiotics Targeting LpxC
Source: mBio. 2017 Jul 25;8(4):e00674-17. doi: 10.1128/mBio.00674-17 (PMC5527309; doi:10.1128/mBio.00674-17)
Supplement: TABLE S3 [file mbo004173392st3.pdf]

Supplementary Table 3. Pharmacokinetics of the LpxC inhibitors LPC-058 and LPC-069

	Pharmacokinetic parameters					
	with liver microsomes		in mice[a]			
Compound	$T_{1/2}$(min)	Intrinsic clearance (µL/min/mg)	AUC (µL/h/mL)	$T_{1/2}$(min)	Plasma clearance (mL/min/kg)	Distribution volume (L/kg)
LPC-058	95	24	21.7	53	15	1.1
LPC-069	44	52	2.5	10	262	3.8

[a] determined after the intravenous injection of 20 mg/kg LPC-058 or 40 mg/kg of LPC-069. $T_{1/2}$: half-life; AUC: area under the curve.
